# Longitudinal Changes in General Overweight and Obesity, and Central Obesity from Birth to Early Adolescence

**DOI:** 10.3390/nu18081206

**Published:** 2026-04-10

**Authors:** Yi Lin, Zeng-Bao Hu, Richard Rankin, Stuart McDonald, Xiao-Yong Li, Feng Wang, Si-Jia Wang, Guo-Lin Bian, Qing-Hai Gong

**Affiliations:** 1 Faculty of Humanities and Social Sciences, University of Nottingham Ningbo China, Ningbo 315100, China; lily.lin@nottingham.edu.cn (Y.L.); sdhy775@126.com (Z.-B.H.); stuart.mcdonald@nottingham.edu.cn (S.M.); 2 Faculty of Science and Engineering, University of Nottingham Ningbo China, Ningbo 315100, China; richard.rankin@nottingham.edu.cn; 3 Yinzhou District Center for Disease Control and Prevention, Ningbo 315100, China; li_xy1115@163.com; 4 Ningbo Municipal Center for Disease Control and Prevention, 1166 Fanjiang’an Road, Ningbo 315100, China; wfcat@126.com (F.W.); 8582021@163.com (S.-J.W.)

**Keywords:** birth weight, body mass index, waist circumference, general obesity, central obesity, childhood, adolescence

## Abstract

Aims: This study aimed to examine the associations between both birth weight (BW) and body mass index (BMI)/waist circumference (WC) measured at ages 7–10 years, and adolescent overweight (OW)/obesity (OB), and central OB at ages 11–13 years. Methods: Longitudinal data were collected from children’s and parents’ questionnaires. Anthropometric data were obtained from health check-ups. BW (kg) was categorized into three groups: <3.0, 3.0–3.9 and ≥4.0 (macrosomia). Underweight (UW)/normal weight (NW), OW and OB were defined based on sex- and age-specific reference values for Chinese children. Central OB was identified using the sex-specific waist-to-height ratio (WHtR) cutoffs. Results: Of the 1204 children, 14.5% had a BW < 3.0 and 15.6% had macrosomia. The rates of OB and central OB were 10.13% and 28.32%, respectively, among children aged 7–10 years and 6.23% and 23.34%, respectively, among those aged 11–13 years. An increasing BW z-score was associated with higher odds of OW/OB in girls aged 11–13 years. Childhood BMI and WC z-scores were associated with higher odds of OW/OB and central OB, respectively, at ages 11–13 years. Childhood OW/OB and central OB were associated with a higher risk of OW/OB and central OB, respectively, at ages 11–13 years. Conclusions: BW was modestly associated with OW/OB in girls. Childhood BMI was the strongest predictor of OW/OB, while childhood WC was a strong and significant predictor of central OB in early adolescence. These findings highlight that early school age is a critical period for risk identification and the implementation of future preventive strategies.

## 1. Introduction

With rapidly increasing rates of general overweight (OW) and obesity (OB) worldwide (henceforth referred to as OW and OB) childhood OW and OB are major global health concerns in the 21st century [1]. Over the past 40 years, the prevalence of OW and OB in children and adolescents worldwide increased from 4% in 1975 to 20% in 2022 [2]. Due to the fast economic boom, urbanization, and transitions in dietary patterns and lifestyles over the past four decades in China, childhood OW and OB have undergone a rapid growth. For Chinese children and adolescents, the prevalence of OW and OB increased from 1% and 0.1%, respectively, in 1985, to 13.8% and 9.6%, respectively, in 2019 [3,4].

Childhood OB is associated with an increased risk of children’s physical and psychological comorbidities, as well as impaired academic performance [5,6]. In addition, childhood OB increases the likelihood of OB persisting into adolescence and adulthood [7]. Thereby, the long-term consequences include chronic diseases, premature death and disability in later life [2,8,9]. Three periods are critical for the development of childhood OW and OB, and central OB which may persist into adulthood: the prenatal period (usually represented by birth weight (BW)), the period of adiposity rebound during childhood (3–7 years), and physiological maturity during adolescence [10]. The prenatal period is significant, according to the Developmental Origins of Health and Disease (DOHaD) hypothesis; nutritional conditions in utero can influence fetal growth and alter the intrauterine environment, thereby leading to variations in BW [11]. BW can be linked to adipose tissue biology, including adipose cell size and the number of adipose cells in later life [12,13,14]. Therefore, BW is associated with body composition including fat mass, body mass index (BMI), and waist circumference (WC), which are known risk factors for OB and central OB later in life, possibly through pathways involving metabolic profiles.

It remains unclear whether BW reliably predicts OW and OB across the lifespan. Epidemiological studies showed that BW was evidently associated with BMI, body composition and the risk of OB in childhood [9,15], adolescence [16,17] and adulthood [18]. Many studies have investigated the association of BW with body weight status in early childhood to predict OW and OB later in life through BMI tracking. Previous cross-sectional and longitudinal studies revealed that macrosomia (≥4.0 kg) was associated with an increased risk of OW and OB in school-age children [17,19,20]. Conversely, low BW (<2.5 kg) has also been linked to higher OB risk in later life, through the mechanism of rapid catch-up growth during early childhood [21]. Evidence regarding the relationship between BW and childhood OW and OB remains inconclusive. However, childhood BMI alone is not an accurate indicator of weight status, health and OB-related morbidity in adolescents and adults [22]. WC is widely used to define central OB and is strongly positively associated with cardiometabolic risk factors including metabolic syndrome, insulin resistance, dyslipidemia, and hypertension, and related chronic diseases [23,24]. A recent review stated that WC is a better and stronger predictor of central OB and WC is considered a key driver of obesity-related health risks and cardiometabolic disorders [25]. Unlike well-studied childhood OW and OB by BMI tracking, little is known on central OB by WC tracking over time.

The differences in developing childhood OW and OB may reflect sex-related variations in pubertal timing, body composition trajectories, hormonal profiles, and health-related behaviors. Previous studies have reported sex-specific differences in the associations of BW, childhood BMI and WC, with adolescent OB and central OB [19,26,27]. From a public health perspective, research on the tracking of BW into childhood and adolescence using both BMI and WC is important for public health specialists and clinicians aiming to prevent the OW epidemic. To our best knowledge, this research topic has not been well studied in China. Several studies have examined the association between BW and either OB [27,28,29,30] or central OB [31] among Chinese infants, children and adolescents. Only one study has simultaneously used both BMI and waist-to-height ratio (WHtR) to assess the relationship between BW and both OB and central OB among Chinese children and adolescents [32]. However, no Chinese study has assessed the longitudinal tracking of both OB and central OB across multiple age groups.

The purpose of this Chinese longitudinal cohort study was to investigate the associations between both BW and childhood BMI/WC, and OW/OB, and central OB in early adolescence. Sex-specific differences in these associations were examined as well. This study contributes to an emerging literature on early-life predictors of adiposity in Chinese children and adolescents and provides scientific support to develop early-life interventions and obesity prevention strategies.

## 2. Methods

### 2.1. Study Design and Population

The Ningbo Youth Risk Behavior Survey (YRBS) was conducted in 10 districts of Ningbo, China from October 2016 to October 2019. The target samples from 22 schools, including nine primary schools and 13 middle schools, were randomly selected. Invitations were sent to school principals and school administrations. With permission, grades and classes were randomly selected in each school for this study.

Eligible participants were children residing in Ningbo, aged 6.5 to 18 years, who provided written informed consent obtained from a parent or legal guardian. Children with a disability or an injury, that could affect the physical examination, were excluded from the survey. In this study, children aged from 7 to 10 years, who participated in the baseline survey (2016), were included. Further methodological details were reported previously [33,34].

**Figure 1:**
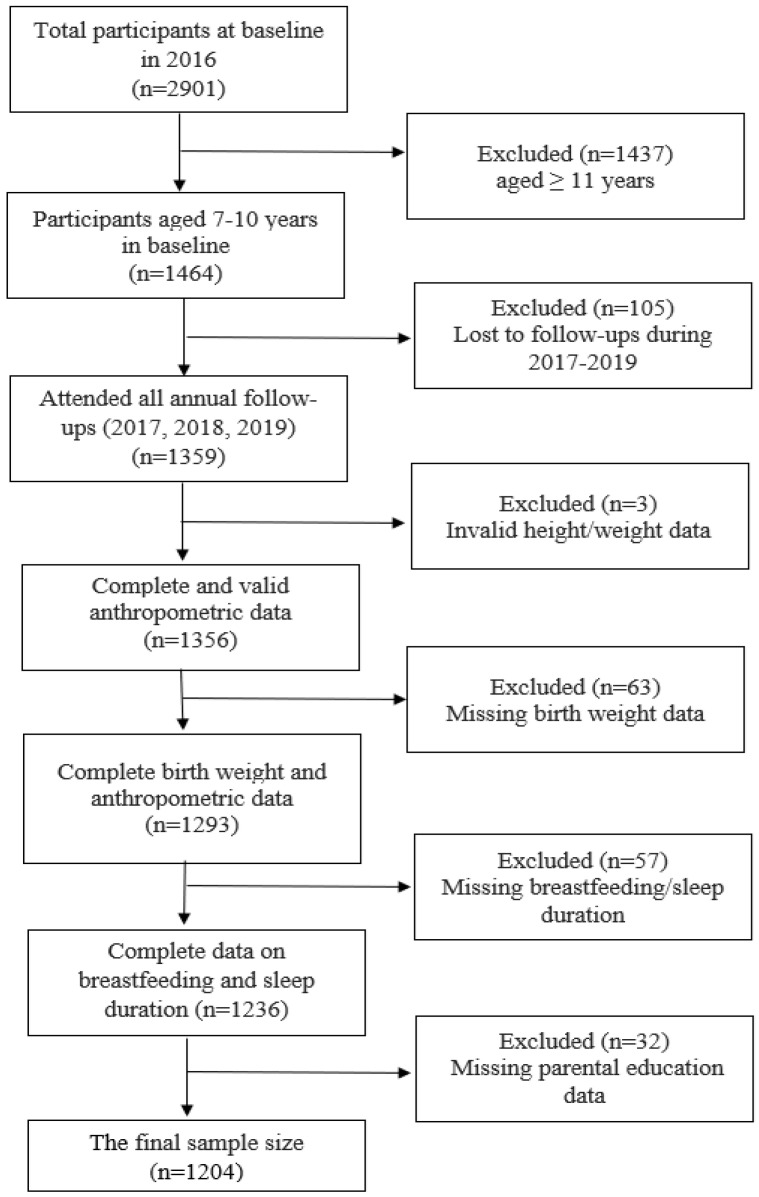
Flowchart of the study population participating in the school-based longitudinal study.

In 2016, of the 2901 children invited to participate (Figure 1), 1437 children were excluded due to not meeting the age criterion. Out of the remaining 1464 children, 105 were lost to follow-up, and 155 were excluded due to missing or invalid data. The final sample size was 1204 children. Of the 1464 children meeting the age criteria at baseline, self-reported weight and height (used to calculate BMI) were available for 1421 (97.06%), and WC data were available for 1402 (95.77%), with 2.94% and 4.23% missing data, respectively. Due to the low proportion of missing data of primary outcomes (<5%), we conducted a complete-case analysis, including only participants with complete data on exposure, outcome, and all covariates in the final models. Therefore, no imputation was performed in our study.

This study was approved by the ethics committee of the Ningbo Center for Disease Control and Prevention (No. 201703) and followed the Declaration of Helsinki. Written informed consent was obtained from all children and their parent or legal guardian.

### 2.2. Questionnaire and Data

In the 2016 baseline survey, all the eligible children were asked to complete a self-administered standardized questionnaire (henceforth referred to as the questionnaire) within one hour during their regular class time under the supervision of well-experienced researchers. Standardized instructions were provided, and assistance was offered uniformly to any child who required clarification on the questions. The questionnaire was developed based on the YRBS in the United States [35]. The questionnaire, covering socio-economic status, demographics, dietary behaviors, lifestyle, and physical and mental health status, has been validated and widely used in school-aged children in Zhejiang Province [33,36]. During follow-up surveys conducted in 2017, 2018 and 2019, children completed the same questionnaires. All data was double-checked for quality control by researchers. Missing or misreported information was re-collected during the survey. In case of significant discrepancies (e.g., sex, birth year), researchers contacted the participants to verify and correct data. For the missing data, children were asked to re-complete those questions if they were willing and able to do so.

Additionally, the children’s parents or legal guardians completed a separate parental questionnaire that collected data on parental education, employment status, child’s birth situation and BW. The details of the questionnaires have been reported previously [33,34].

Parent-reported BW (kg) was collected through a parental questionnaire during the follow-up surveys. BW z-score was calculated using World Health Organization (WHO) reference data [37]. Given the low proportion of children with BW < 2.5 kg (2.6%), BW was categorized into three groups: <3.0, 3.0–3.9 and ≥4.0 (macrosomia) based on Chinese reference data [38]. Data on breastfeeding was also obtained from parental reports. Exclusive breastfeeding duration (months) was classified into four groups, based on the recommendation issued by the WHO and United Nations Children’s Fund (UNICEF) [39]: none, 1–5, 6 and ≥7.

Parents or guardians of each child were asked about their education level. The highest degree of maternal and paternal education levels was categorized into three levels: no formal education or lower than secondary education, secondary education and higher education (bachelor’s degree or higher). Family structure, as reported by children, was recorded into three categories: nuclear family, single-parent family and others (e.g., joint family, extended family).

### 2.3. Anthropometric Measurements

At each survey wave, all children underwent a standardized physical examination conducted by trained medical professionals in the early morning while in a fasting state at their schools, followed by the questionnaire section. Anthropometric measurements were obtained once for each child following a standardized protocol [40]. If a measurement was suspected to be erroneous, it was repeated immediately, and the second reading was used. Consistent measurement protocols and calibrated equipment were used across all sites, as described previously [33,34].

To ensure data quality, 5% of children were randomly selected for re-measurement on the same day by the same or another trained medical professional. The measurement was accepted if the difference between the two readings did not exceed 0.1 kg for weight or 0.5 cm for height. The daily anthropometric data were considered valid if the overall error rate from re-measurements among all re-measured children did not exceed 5%. When this criterion was met, data quality was affirmed.

Body weight was measured to the nearest 0.1 kg using an electronic scale and height was measured to the nearest 0.1 cm using a free-standing stadiometer. WC was measured at the midpoint between the inferior costal margin and the iliac crest along the midaxillary line.

BMI, calculated as weight (kg)/height^2^ (m^2^), was used to identify OW and OB. BMI z-score was calculated using WHO reference data [41]. Chinese children’s weight status was classified into underweight (UW)/normal weight (NW), OW and OB, using sex- and age- specific reference data from the National Health and Family Planning Commission of China [42].

WC is a reliable measure of abdominal fat accumulation and is widely used to diagnose central OB [28]. WC z-score was calculated using the LMS method based on NHANES III reference data [43]. In addition, waist-to-height ratio (WHtR) was calculated as WC (cm)/height (cm). Based on the guideline proposed by the Chinese Medical Association [44], central OB was defined by sex-specific cut-off values of WHtR (boys: ≥0.48 and girls ≥0.46), which reflect sex-specific differences in abdominal fat distribution and cardiometabolic risk.

### 2.4. Statistical Analysis

Descriptive statistics were presented using number and percentage for category variables and mean with standard deviation (SD) for continuous variables. Normality of continuous variables was tested by Shapiro–Wilk and Shapiro–Francia tests and a histogram. Statistical differences in mean values and percentages between boys and girls were compared by Student’s *t*-test and a chi-square (Χ^2^) test, respectively.

A sensitivity analysis was undertaken for comparison of baseline anthropometric data, including BW, BMI and WC, between the dropout participants aged 7–10 years and all the participants aged 7–10 years in the final analysis, to detect whether values of dropouts affect the outcomes.

Scatterplots were used to examine the curve shape between baseline BW/BMI/WC at ages 7–10 years (2016) and BMI/WC at ages 11–13 years (2019). A linear relationship was observed.

To account for the correlation between the repeated anthropometric measurements (baseline and follow-up in 2019) from the same participant, the associations between the status of OW and OB/central OB in children aged 11–13 years, and independent variables were examined using Generalized Estimating Equations (GEE) with a binomial distribution, a logit link function, and an exchangeable correlation structure. Associations were investigated via two models: (1) a crude model; (2) a multivariable model: adjusting for child’s sex, age, breastfeeding status, night sleep duration (11–13 years), and physical activity level (11–13 years), as well as the highest educational degree of both parents. Additional GEE with a Poisson distribution were used to estimate relative risk (RR). Each independent variable, whether a continuous or categorical variable, was analyzed in separate crude or multivariable models. In addition, continuous independent variables (BW z-score, BMI z-score, WC z-score) were included in the models as linear terms, while categorical independent variables were coded as dummy variables with the first category serving as the reference group. Multicollinearity among independent variables was evaluated by variance inflation factors (VIF). Interaction between sex and exposure was tested to examine potential effect modification by sex (Supplement Table S3). 

Results were considered statistically significant at a two-tailed level of 0.05. Statistical analysis was conducted using the STATA statistical software package version 19 (2021).

## 3. Results

The results of the sensitivity analysis showed no significant differences in baseline BW, BMI and WC between all participants and dropout participants (BW: *p* = 0.409, BMI: *p* = 0.057, WC: *p* = 0.108).

### 3.1. Children’s Characteristics and Anthropometric Measures at Birth, Childhood (7–10 Years) and Early Adolescence (11–13 Years)

A total of 1204 children aged 7–10 years (mean age of 8.68 years) participated in the baseline survey (Table 1). Over half of the children (50.66%) were breastfed for longer than 6 months, and 43.85% engaged in moderate-to-vigorous physical activity on more than five days per week. In addition, 37.04% of mothers and 37.96% of fathers had attained higher education.

The average BW of the total children was 3.54 kg with BMI z-score of 0.42 (Table 2). Mean values for body weight, BMI, WC and WHtR were 29.34, 16.51, 59.92 and 0.45, respectively, among children aged 7–10 years; the values were 40.39, 18.06, 65.18 and 0.44, respectively, among those aged 11–13 years. Boys aged 7–10 and 11–13 years had significantly higher mean anthropometric values than girls of the same age groups.

### 3.2. Longitudinal Patterns of Adiposity from Birth Weight Through Childhood to Early Adolescence

About 14.45% of children had a BW < 3.0 kg and around 15.61% of children were macrosomia with the higher proportion of BW < 3.0 in girls and macrosomia in boys (Table 2). Among children aged 7–10 years, the prevalence of OW, OB and central OB were 10.13%, 10.38% and 28.32%, respectively.

**Figure 2:**
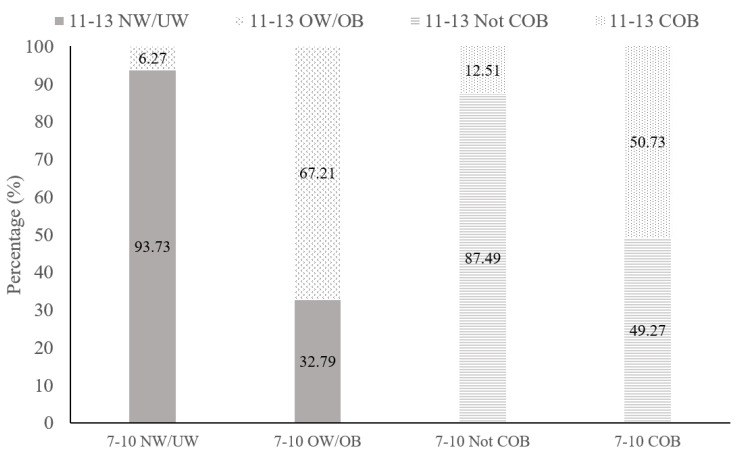
Tracking of body weight status from childhood (ages 7–10 years) to adolescence (ages 11–13 years). Abbreviations: COB: central obesity, NW: normal weight, OB: obesity, OW: overweight, UW: underweight.

The tracking of OW and OB from childhood (7–10 years) to early adolescence (11–13 years) is shown in Figure 2. Among children aged 7–10 years classified as OW or OB, 67.2% remained in the same weight category among those aged 11–13 years. In contrast, only 6.27% of those aged 7–10 years with UW or NW developed new-onset OW or OB among those aged 11–13 years. Regarding central OB, 50.7% of children with central OB at 7–10 years remained so aged 11–13 years, whereas 87.5% of those non-central obese children aged 7–10 years continued to be non-centrally obese.

### 3.3. Anthropometric Characteristics Stratified by Body Weight Status from Birth to Early Adolescence (11–13 Years)

Differences in anthropometric values from birth throughout later childhood and early adolescence stratified by body weight status are presented in Table 3. OW and obese girls aged 11–13 years had higher mean BW compared to UW and NW counterparts. From late childhood (7–10 years) to early adolescence (11–13 years), the mean differences in body weight, BMI, WC, and WHtR between the OW and OB group, and the UW and NW group increased markedly. These differences were consistent in both sexes but were slightly more pronounced among girls than boys. All observed differences across weight status groups were statistically significant (*p* < 0.001).

### 3.4. The Relationship Between Birth Weight and Overweight, Obesity and Central Obesity in Early Adolescence (11–13 Years)

A higher BW z-score was modestly associated with an increased likelihood of OW and OB in girls (OR = 1.22, 95% CI: 1.06, 1.40), whereas no significant association was observed in the total sample and boys (Table 4). In addition, BW categories and BW z-score were not significantly associated with central OB.

### 3.5. The Relationship Between Childhood Overweight and Obesity, and Central Obesity in Early Adolescence (11–13 Years)

In the multivariable adjusted model, higher BMI z-score and WC z-score in later childhood (7–10 years) were associated with increased odds of developing OW and OB, and central OB, respectively, in the total sample, and both boys and girls in early adolescence (11–13 years). Children with OW and OB at ages 7–10 years were likely to remain in the OW and OB category at ages 11–13 years compared to their UW/NW peers: with 32-fold higher odds in the total sample (OR = 32.66, 95% CI: 21.95, 48.61, RR: 10.17), 30-fold higher in boys (OR = 30.33, 95% CI: 18.24, 50.46, RR = 7.91), and 46-fold higher in girls (OR = 46.09, 95% CI: 22.73, 93.46, RR: 17.06) at ages 11–13 years (Table 4). At the same time, children with central OB had about eightfold (OR = 8.05, 95% CI: 5.92, 10.94, RR: 4.12), ninefold (OR = 9.59, 95% CI: 6.20, 14.83, RR: 4.13) and sevenfold (OR = 7.19, 95% CI: 4.56, 11.33, RR: 4.15) increased odds of developing central OB in total, boy and girl adolescents, respectively, at ages 11–13 years (Table 5, Supplement Tables S1 and S2).

## 4. Discussion

This school-based longitudinal study revealed that increasing BW z-score was only significantly associated with higher odds of OW and OB among girls aged 11–13 years. Tracking of both BMI and WC from late childhood to early adolescence indicated strong associations. Children with OW and OB, or central OB showed a significantly higher likelihood of maintaining OW and obese status, as well as central OB in early adolescence.

This study reports that both the mean BW and the prevalence of macrosomia were higher than those reported in previous cross-sectional studies among Chinese children [28,32,45]. The discrepancy, most likely, reflects China’s ongoing nutritional transition, which has steadily affected maternal feeding culture and diets during pregnancy, thereby influencing fetal growth and BW outcomes [46]. The prevalence of OW and OB among children aged 11–13 years in our study was slightly higher than that reported in 2022 among Ningbo adolescents aged 12–19 years from recent studies (16.6%) and close to the estimate in 2019 for children aged 3–18 years in eastern China (18.2%) [47,48,49], but relatively lower than the national prevalence of 23.4% in 2019 among Chinese adolescents aged 7–18 years [3]. Compared to foreign studies, our OW and OB prevalence was lower than the overall OW prevalence reported in European children aged 3–16 years in 2025 (21.6%), but was comparable to the European OB prevalence (7.0%) [50]. In contrast, U.S. national data show substantially higher childhood OB prevalence, ranging from 13.9% to 15.2% in children aged 5–9 years and 18.6% to 24.6% in children aged 10–14 years across both sexes [51]. Notably, the prevalence of central OB in our study among children aged 7–10 and 11–13 years was much higher than that of Chinese children from previous studies [31,32], but still lower than that reported in Greek school-aged children (33.4%) [52].

It is well-known that BW is a risk factor for OB during childhood and adolescence [53]. Previous studies suggested that both low BW (<2.5 kg) and higher BW (≥3.0 kg) were associated with an elevated risk of OW and OB [29,32] and central OB [31,32] in Chinese children and adolescents. Extensive studies on the association between BW and the subsequent development of OB and central OB in later life are controversial. Results from large population-based studies showed a J-shaped association and a U-shaped association of BW with BMI and WHtR in children and adolescents [26,31]. In a Chinese longitudinal study, higher BW status was associated with an increased risk of OW and OB in 15,852 Chinese children aged 3–6 years [45]. In contrast, BW status was not associated with the risk of OW and OB, and central OB in adolescents aged 11–13 years in our study, which is inconsistent with previous studies in school-age children [17,31,54,55]. This discrepancy may be attributable to: (1) the low prevalence of BW and macrosomia in our study, reducing statistical power to detect associations across the full BW categories; (2) the potential effect of postnatal growth patterns, which was not collected in this study.

Rapid weight gain in early childhood may be related to increased risks of developing OB and cardiometabolic diseases later in life [20,56,57]. Our results suggested that high BMI (OR: 7.78, 95% CI: 5.96, 10.17) in childhood were the strongest predictors of OW and OB in early adolescence. In line with our results, previous longitudinal studies have reported substantial tracking of BMI and weight status from early childhood (ages 5–7 years) into late adolescence [19,58]. A population-based longitudinal study showed that severe OW and OB in Norwegian children aged 5–7 years were more strongly associated with OW and OB in Norwegian adolescents aged 15–20 years (OR: 11.51) [19]. A systematic review of 21 studies concluded that rapid weight gain before the preschool period (up to 3 years), infancy in particular, is associated with subsequent OB risk [57]. Similar reports from a Finnish longitudinal study revealed that BMI at 6 months was only significantly correlated with BMI at age 7, but not with BMI at age 15, while BMI at age 7 was significantly correlated with BMI at age 15 [58]. The possible mechanism is that dietary and lifestyle behaviors formed during a critical development period of childhood, which may have a greater effect on OW phenotype development compared to those established in infancy.

Beyond BMI as a surrogate indicator of overall fat mass, WC can provide an additional and independent predictive value for health risk [2,25]. Our results indicate that WC (OR: 4.86, 95% CI: 3.89, 6.08) is a better and strong predictor of central OB and cardiometabolic risks, compared to BMI [25]. The available literature for tracking of WC and central OB from childhood to adolescence is limited in China. In agreement with the reports from other longitudinal studies assessing similar age groups, more than half of children aged 7–10 years with central OB kept the same status of central OB in adolescence and abdominal fat distribution may be determined as early as age 7 [59,60]. Therefore, interventions aimed at childhood OB prevention, particularly central OB should be initiated before 7 years.

Notably, our results show that BW is a modest predictor of later OW and OB (OR: 1.22, 95% CI: 1.06, 1.40), rather than central OB (OR: 1.12, 95% CI: 0.98, 1.26), among girls aged 11–13 years. In addiiton, a higher proportion of girls than boys in our study were categorized as OW or OB, and central OB in childhood (ages 7–10 years). However, the prevalence of OW and OB, and central OB were higher in boys. This is consistent with the findings from several longitudinal studies [5,16,19,61]. Sex differences may be partly explained by biological factors. This is attributable to greater fat mass and less fat-free mass in girls after birth [62]. In addition, girls generally experience pubertal onset earlier than boys, which results in changes in body composition. Moreover, increasing levels of sex steroid hormones during late childhood play a key role in fat distribution and lean mass accumulation [62].

The implications of our findings extend beyond the study population and transcend geographical boundaries, contributing to the global evidence on early-life determinants of OB and central OB. Given the alarming increase in OB, and central OB, this study addresses a critical gap in the international literature by identifying population-specific risks such as BW, BMI, and WC derived from our large Chinese longitudinal cohort study. Importantly, our findings can provide practical insights for public health practitioners to drive effective public health actions. First, WC measurements should be incorporated into standard pediatric health check-ups. Second, routine monitoring of both BMI and WC is a key strategy for early identification of cardiometabolic risk. Third, school- and community-based interventions should be implemented to promote healthy lifestyles. Therefore, these life-course strategies for early OB prevention are crucial to mitigate the rising global epidemic of childhood adiposity.

This longitudinal study was the first study to track OW and OB, and central OB from birth to early adolescence in Zhejiang Province using standardized children’s and parental questionnaires along with health checks. Nevertheless, this study has several limitations. First, children’s BW was obtained via a parental questionnaire when children were aged 7–10 years, rather than hospital birth records, thus relying on parental recall. Although this may introduce some degree of recall bias, previous studies demonstrated good accuracy between parental recall of BW and medical records, even over ten years after delivery [63,64]. Second, we did not collect data on birth length or growth patterns from infancy to early childhood. These factors are known to influence the risk of OB and central OB in later childhood and adolescence [65]. Future studies could address this by linking cohort data with electronic maternal and child health records from community health centers or applying multiple imputation techniques where auxiliary variables are available to estimate missing early-life data. Third, our models did not adjust for certain lifestyle factors such as diet quality and screen time, which might be potential confounders in the associations. Although key confounders including sociodemographic and behavioral confounders were controlled, the possibility of residual confounding from these unmeasured variables cannot be excluded. Fourth, since PA and sleep were measured concurrently with the outcomes, they were treated as potential confounders, rather than mediators, in our models. The distinction between confounders and mediators remains challenging in observational studies, and future studies with longitudinal measurements of PA and sleep are needed to test mediation. Another limitation is that the pubertal stage (e.g., Tanner stage) was not assessed, which is a key determinant of body weight and body composition during childhood and adolescence. Therefore, the absence of the pubertal stage may have influenced the observed associations. Moreover, our study lacked data on parental anthropometric and health characteristics during pregnancy, including paternal weight, maternal gestational weight gain, paternal health status, nutritional status, cardio-metabolic conditions, as well as early childhood growth trajectories of participants. Since these multiple factors are associated with fetal growth and long-term risk of OB, they may confound the observed associations. Finally, our analysis may be subject to mathematical coupling as both the exposure (e.g., childhood BMI) and the outcome (follow-up overweight/obesity defined by BMI) are derived from shared anthropometric components (weight and height). This likely contributed to the large effect size. However, the tracking of adiposity status from childhood into adolescence aligns with the well-documented phenomenon of body weight status tracking during adolescence [5,16,19,61]. Future well-designed longitudinal studies with more frequent measurements, and comprehensive collection of perinatal, childhood, and adolescent covariates, along with assessment of body composition from birth to adulthood are needed to better understand the full trajectory of OB development from infancy to late adolescence.

Although our study focused on the tracking of body weight and WC, it is worth noting that persistent high body weight or large WC at birth or in childhood may be associated with adverse cardiometabolic outcomes later in life, such as elevated blood pressure and dyslipidemia, thereby highlighting the clinical relevance of early adiposity trajectories.

## 5. Conclusions

This is the first longitudinal study in Zhejiang Province to examine the co-trajectories of general and central adiposity from birth to early adolescence in a large, school-based cohort, thereby strengthening the generalizability of our findings for childhood OB prevention in eastern China. Our results reveal that an increase in BW z-score, but not BW categories, was modestly associated with a higher likelihood of OW and OB among girls in early adolescence. In this Chinese longitudinal study, childhood BMI at ages 7–10 years was the strongest predictor of later OW and OB, while WC was a strong and significant predictor of central OB in early adolescence. This study highlights the value of longitudinally tracking both BMI and WC across the childhood-to-adolescence transition, providing important insights into their predictive values for later adiposity in Chinese youth.

While our findings are robust, certain limitations such as reliance on parental recall of BW and the lack of detailed growth data from infancy to early childhood should be considered. Importantly, our results indicate that adiposity status during early school age (7–10 years) is a key predictor of both general and central obesity in adolescence, highlighting the importance of early monitoring and preventive efforts before this critical period. These findings have implications for both short-term and long-term strategies. In the short term, family- and school-based programs should promote healthy lifestyles to reduce the risk of later-onset adiposity. Meanwhile, long-term strategies should establish life-course health surveillance systems integrating maternal, infant, childhood and adolescent health data to support at-risk children from infancy onward.

## Figures and Tables

**Table 1:** Characteristics of children aged 11–13 years.

Characteristics	Total (n = 1204)	Boys (n = 630)	Girls (n = 574)	*p* *
	n (%)			
**11–13 Years**				
Paternal education				0.187
No formal education or lower than secondary education	417 (34.63)	233 (36.98)	184 (32.06)	
Secondary education	330 (27.41)	164 (26.03)	166 (28.92)	
College or above degree	457 (37.96)	233 (36.98)	224 (39.02)	
Maternal education				0.122
No formal education or lower than secondary education	445 (36.96)	250 (39.68)	195 (33.97)	
Secondary education	313 (26.00)	156 (24.76)	157 (27.35)	
College or above degree	446 (37.04)	224 (35.56)	222 (38.68)	
Breastfeeding				0.521
No	120 (9.97)	70 (11.11)	50 (8.71)	
1–5 months	295 (24.50)	154 (24.44)	141(24.56)	
6 months	179 (14.87)	89 (14.13)	90 (15.68)	
≥7 months	610 (50.66)	317 (50.32)	293 (51.05)	
30 min moderate-to-vigorous physical activity				0.080
No	77 (6.40)	50 (7.94)	27 (4.70)	
1–2 days	247 (20.51)	122 (19.37)	125 (21.78)	
3–4 days	352 (29.24)	176 (27.94)	176 (30.66)	
5–7 days	528 (43.85)	282 (44.76)	246 (42.86)	
	Mean (SD)			
Sleep duration (11–13 years)	9.65 (0.76)	9.67 (0.80)	9.63 (0.72)	0.330
Age of children aged 7–10 years (years)	8.68 (0.38)	8.68 (0.40)	8.67 (0.36)	0.595
Age of children aged 11–13 years (years)	11.68 (0.38)	11.68 (0.40)	11.67 (0.36)	0.596

SD: standard deviation * Chi-square (Χ^2^) test was used to examine the difference for category groups; Student’s *t*-test was used to examine the difference between boys and girls for continuous variables.

**Table 2:** Characteristics and classification of birth weight, body weight and waist circumference from birth to early adolescence.

	Total	Boys	Girls	*p* *
**Birth**				
Birth weight (kg)	3.54 (0.93)	3.61 (1.03)	3.46 (0.79)	0.006
Birth weight z-score	0.42 (1.70)	0.43 (1.82)	0.42 (1.56)	0.866
Birth weight status				0.003
<3.0 kg	174 (14.45)	71 (11.27)	103 (17.94)	
3.0–3.9 kg	842 (69.93)	451 (71.59)	391 (68.12)	
≥4.0 kg	188 (15.61)	108 (17.14)	80 (13.94)	
**7–10 years**				
Height (cm)	132.81 (6.21)	133.16 (6.08)	132.43 (6.33)	0.039
Weight (kg)	29.34 (6.26)	30.21 (6.56)	28.38 (5.77)	<0.001
Weight z-score	3.66 (0.48)	3.74 (0.43)	3.56 (0.52)	<0.001
BMI (kg/m^2^)	16.51 (2.57)	16.91 (2.69)	16.08 (2.35)	<0.001
BMI z-score	−0.01 (0.99)	0.14 (1.04)	−0.18 (0.91)	<0.001
Waist circumference (cm)	59.92 (7.08)	60.96 (7.49)	58.78 (6.42)	<0.001
Waist circumference z-score	−0.124 (0.99)	0.03 (1.00)	−0.29 (0.95)	<0.001
Waist-to-height-ratio	0.45 (0.05)	0.46 (0.05)	0.44 (0.04)	<0.001
Body weight status				0.006
Underweight and normal weight	957 (79.49)	479 (76.03)	478 (83.28)	
Overweight	122 (10.13)	72 (11.43)	50 (8.71)	
Obesity	125 (10.38)	79 (12.54)	46 (8.01)	
Central obesity				0.065
No	863 (71.68)	466 (73.97)	397 (69.16)	
Yes	341 (28.32)	164 (26.03)	177 (30.84)	
**11–13 years**				
Height (cm)	149.10 (7.75)	148.38 (7.74)	149.89 (7.68)	<0.001
Weight (kg)	40.39 (9.19)	40.96 (9.51)	39.77 (8.79)	0.025
Weight z-score	3.56 (0.79)	3.88 (0.74)	3.20 (0.68)	<0.001
BMI (kg/m^2^)	18.06 (3.33)	18.48 (3.43)	17.60 (3.16)	<0.001
BMI z-score	−0.01 (0.99)	0.11 (1.01)	−0.15 (0.94)	<0.001
Waist circumference (cm)	65.18 (8.66)	66.50 (9.14)	63.74 (7.86)	<0.001
Waist circumference z-score	−0.29 (1.07)	−0.16 (1.12)	−0.43 (0.99)	<0.001
Waist-to-height ratio	0.44 (0.05)	0.45 (0.06)	0.43 (0.05)	<0.001
Body weight status				<0.001
Underweight and normal weight	978 (81.23)	475 (75.40)	503 (87.63)	
Overweight	151 (12.54)	1104 (16.51)	47 (8.19)	
Obesity	75 (6.23)	51 (8.10)	24 (4.18)	
Central obesity				0.077
No	923 (76.66)	470 (74.60)	453 (78.92)	
Yes	281 (23.34)	160 (25.40)	121 (21.08)	

BMI: body mass index. * *p* for category variables was examined using a chi-square (Χ^2^) test and *p* for the difference between boys and girls for continuous variables was examined using Student’s *t*-test.

**Table 3:** Anthropometric characteristics stratified by body weight status from birth to early adolescence (11–13 years).

	Total					Boys					Girls				
	Normal and Underweight (n = 978)		Overweight and Obesity (n= 226)		*p* *	Underweight and Normal Weight (n = 475)		Overweight and Obesity (n = 155)		*p* *	Underweight and Normal Weight (n = 503)		Overweight and Obesity (n = 71)		*p* *
	Mean	95% CI	Mean	95% CI		Mean	95% CI	Mean	95% CI		Mean	95% CI	Mean	95% CI	
**Birth**															
Birth weight (kg)	3.50	3.50, 3.60	3.60	3.50, 3.80	0.087	3.60	3.50, 3.70	3.60	3.50, 3.80	0.963	3.40	3.40, 3.50	3.70	3.40, 3.90	0.013
Birth weight z-score	0.39	0.28, 0.49	0.58	0.35, 0.81	0.129	0.42	0.25, 0.59	0.46	0.21, 0.71	0.840	0.36	0.23, 0.48	0.84	0.37, 1.32	0.013
**7–10 years**															
Weight (kg)	27.63	27.33, 27.92	36.74	35.84, 37.65	<0.001	28.13	27.69, 28.57	36.56	35.46, 37.66	<0.001	27.15	26.77, 27.53	37.14	35.50, 38.78	<0.001
BMI (kg/m^2^)	15.75	15.64, 15.86	19.82	19.45, 20.19	<0.001	15.95	15.80, 16.11	19.84	19.37, 20.31	<0.001	15.56	15.40, 15.72	19.79	19.18, 20.39	<0.001
BMI z-score	−0.31	−0.35, −0.27	1.26	1.12, 1.41	<0.001	−0.23	−0.29, −0.17	1.27	1.09, 1.45	<0.001	−0.38	−0.44, −0.32	1.25	1.02, 1.48	<0.001
WC (cm)	58.04	57.70, 58.38	68.07	67.06, 69.07	<0.001	58.53	58.04, 59.02	68.42	67.15, 69.69	<0.001	57.57	57.10, 58.04	67.30	65.67, 68.92	<0.001
WC z-score	−0.36	−0.42, −0.31	0.90	0.80, 1.00	<0.001	−0.27	−0.35, −0.20	0.94	0.82, 1.07	<0.001	−0.44	−0.52, −0.37	0.82	0.65, 0.98	<0.001
WHtR	0.44	0.44, 0.44	0.50	0.49, 0.51	<0.001	0.44	0.44, 0.45	0.51	0.50, 0.51	<0.001	0.44	0.43, 0.44	0.49	0.48, 0.50	<0.001
**11–13 years**															
Weight (kg)	37.41	37.02, 37.80	53.32	52.17, 24.47	<0.001	37.16	36.62, 37.71	52.60	51.22, 53.98	<0.001	37.64	37.08, 38.20	54.88	52.81, 56.95	<0.001
BMI (kg/m^2^)	16.90	16.70, 17.00	23.30	22.90, 23.70	<0.001	17.00	16.80, 17.10	23.20	22.70, 23.60	<0.001	16.80	16.60, 16.90	23.60	22.80, 24.40	<0.001
BMI z-score	−0.37	−0.41, −0.34	1.54	1.42, 1.66	<0.001	−0.34	−0.39, −0.29	1.55	1.37, 1.64	<0.001	−0.40	−0.45, −0.35	1.63	1.38, 1.88	<0.001
WC (cm)	62.68	62.28, 63.07	76.02	74.82, 77.22	<0.001	63.28	62.71, 63.85	76.34	74.86, 77.83	<0.001	62.11	61.57, 62.65	75.30	73.25, 77.36	<0.001
WC z-score	−0.55	−0.61, −0.49	0.85	0.74, 0.96	<0.001	−0.49	−0.58, −0.40	0.88	0.74, 1.01	<0.001	−0.60	−0.68, −0.52	0.79	0.61, 0.97	<0.001
WHtR	0.42	0.42, 0.42	0.50	0.50, 0.51	<0.001	0.43	0.43, 0.43	0.51	0.50, 0.52	<0.001	0.43	0.42, 0.43	0.49	0.48, 0.51	<0.001

BMI: body mass index; CI: confidence interval; WC: waist circumference; WHtR: waist-to-height ratio. * Mean values between underweight/normal weight and overweight/obesity were examined using Student’s *t*-test.

**Table 4:** Crude and adjusted odds ratios for overweight and obesity in early adolescence (11–13 years) using Generalized Estimating Equations.

Overweight and General Obesity																								
	Total								Boys								Girls							
	Crude			Adjusted *					Crude			Adjusted *					Crude			Adjusted *				
	OR	95% CI	*p*	OR	95% CI	*p*	RR	VIF	OR	95% CI	*p*	OR	95% CI	*p*	RR	VIF	OR	95% CI	*p*	OR	95% CI	*p*	RR	VIF
**Birth**																								
Birth weight																								
<3.0 kg	1			1					1			1					1			1				
3.0–3.9 kg	0.68	0.43, 1.09	0.106	0.71	0.44, 1.14	0.155	0.76	1.23	0.82	0.45, 1.51	0.524	0.8	0.43, 1.51	0.503	0.85	1.17	0.67	0.32, 1.41	0.29	0.61	0.28, 1.32	0.207	0.65	1.3
≥4.0 kg	1.23	0.84, 1.80	0.298	1.16	0.78, 1.72	0.465	1.12	1.24	1.12	0.69, 1.81	0.63	1.04	0.64, 1.70	0.865	1.03	1.27	1.35	0.70, 2.63	0.372	1.54	0.77, 3.05	0.211	1.44	1.23
Birth weight z-score	1.06	0.98, 1.15	0.131	1.06	0.98, 1.14	0.177	1.04	1.07	1.01	0.92, 1.11	0.839	0.99	0.90, 1.10	0.92	1	1.08	1.18	1.03, 1.34	0.017	1.22	1.06, 1.40	0.005	1.17	1.09
**7–10 years Weight status**																								
Underweight and normal weight	1			1					1			1					1			1				
Overweight and Obesity	30.64	21.11, 44.47	<0.001	32.66	21.95, 48.61	<0.001	10.17	1.29	27.44	17.04, 44.17	<0.001	30.33	18.24, 50.46	<0.001	7.91	1.34	38.73	20.39, 73.59	<0.001	46.09	22.73, 93.46	<0.001	17.06	1.24
BMI z-score	7.35	5.71, 9.45	<0.001	7.78	5.96, 10.17	<0.001	2.04	1.07	6.84	4.96, 9.44	<0.001	7.49	5.31, 10.57	<0.001	1.93	1.07	8.31	5.44, 12.68	<0.001	9.15	5.82, 14.38	<0.001	2.94	1.09

BMI: body mass index; OR: odds ratio; CI: confidence interval; RR: relative risk; VIF: variance inflation factors. * The model adjusted for child’s breastfeeding status, sex (for total samples), age, area of residence, the highest degree of parental education, child’s duration of sleep at night at 11–13 years, physical activity level at 11–13 years.

**Table 5:** Crude and adjusted odds ratios for central obesity in early adolescence (11–13 years) using Generalized Estimating Equations.

Central Obesity																								
	Total								Boys								Girls							
	Crude			Adjusted *					Crude			Adjusted *					Crude			Adjusted *				
	OR	95% CI	*p*	OR	95% CI	*p*	RR	VIF	OR	95% CI	*p*	OR	95% CI	*p*	RR	VIF	OR	95% CI	*p*	OR	95% CI	*p*	RR	VIF
**Birth**																								
Birth weight																								
<3.0 kg	1			1					1			1					1			1				
3.0–3.9 kg	0.89	0.60, 1.32	0.565	0.89	0.60, 1.34	0.593	0.92	1.23	1.11	0.63, 1.95	0.728	1.09	0.61, 1.96	0.768	1.06	1.17	0.77	0.44, 1.36	0.372	0.77	0.73, 1.38	0.384	0.82	1.30
≥4.0 kg	1.13	0.78, 1.63	0.511	1.12	0.77, 1.62	0.563	1.08	1.24	1.11	0.63, 1.95	0.665	1.04	0.64, 1.71	0.874	1.03	1.27	1.14	0.64, 2.01	0.655	1.25	0.70, 2.25	0.450	1.19	1.23
Birth weight z-score	1.07	0.99, 1.15	0.083	1.07	0.99, 1.15	0.078	1.05	1.07	1.06	0.96, 1.16	0.251	1.05	0.96, 1.16	0.299	1.03	1.08	1.09	0.96, 1.22	0.183	1.12	0.98, 1.26	0.087	1.09	1.09
**7–10 years Central obesity status**																								
No	1			1					1			1					1			1				
Yes	7.20	5.37, 9.65	<0.001	8.05	5.92, 10.94	<0.001	4.12	1.41	8.49	5.66, 12.75	<0.001	9.59	6.20, 14.83	<0.001	4.13	1.39	6.49	4.20, 10.01	<0.001	7.19	4.56, 11.33	<0.001	4.15	1.48
Waist circumference z-score	4.60	3.71, 5.69	<0.001	4.86	3.89, 6.08	<0.001	2.58	1.07	5.36	3.93, 7.31	<0.001	6.08	4.35, 8.49	<0.001	2.71	1.05	4.09	3.01, 5.56	<0.001	4.32	3.14, 5.94	<0.001	2.53	1.14

OR: odds ratio; CI: confidence interval; RR: relative risk; VIF: variance inflation factors. * The model adjusted for child’s breastfeeding status, sex (for total samples), age, area of residence, the highest degree of parental education, child’s duration of sleep at night at 11–13 years, physical activity level at 11–13 years.

## Data Availability

The data is not publicly available due to privacy or ethical restrictions. If there is a reasonable request, it can be obtained from the corresponding authors.

## Supplementary Materials

The following supporting information can be downloaded at: https://www.mdpi.com/article/10.3390/nu18081206/s1, Table S1: Crude and adjusted risk ratios for overweight and obesity in early adolescence (ages 11–13 years) using GEE with a Poisson distribution; Table S2: Crude and adjusted risk ratios for central obesity in early adolescence (ages 11–13 years) using GEE with a Poisson distribution; Table S3: Adjusted odds ratios for overweight/obesity and central obesity in early adolescence (11–13 years) in total adolescents, with interaction by sex, using Generalized Estimating Equations.

## Abbreviations

BW: birth weight; BMI: body mass index; GLM: generalized linear model; NW: normal weight; OB: obesity; OW: overweight; SD: standard deviation; UW: underweight; WC: waist circumference; WHtR: waist-to-height ratio; YRBS: Youth Risk Behavior Survey.
