# Supplementary material for: Longitudinal Changes in General Overweight and Obesity, and Central Obesity from Birth to Early Adolescence
Source: Nutrients. 2026 Apr 10;18(8):1206. doi: 10.3390/nu18081206 (PMC13118507; doi:10.3390/nu18081206)
Supplement: Supplementary file 1 [file nutrients-18-01206-s001.zip › nutrients-4196681-supplementary.pdf]

Supplement Table S1. Crude and adjusted risk ratios for overweight and obesity in early adolescence (ages 11–13 years) using GEE with a Poisson distribution

	Overweight and General Obesity																				
	Total							Boys							Girls						
	Crude			Adjusted*				Crude			Adjusted*				Crude			Adjusted*			
	RR	95% CI	P	RR	95% CI	P	VIF	RR	95% CI	P	RR	95% CI	P	VIF	RR	95% CI	P	RR	95% CI	P	VIF
**Birth**																					
Birth weight																					
<3.0 kg	1			1				1			1				1			1			
3.0-3.9 kg	0.73	0.49, 1.08	0.114	0.76	0.51, 1.12	0.165	1.23	0.86	0.53, 1.38	0.531	0.85	0.53, 1.37	0.504	1.17	0.70	0.35, 1.37	0.297	0.65	0.33, 1.28	0.208	1.30
≥4.0 kg	1.18	0.87, 1.59	0.292	1.12	0.51, 1.12	0.476	1.24	1.09	0.81, 1.38	0.627	1.03	0.72, 1.46	0.869	1.27	1.30	0.74, 2.28	0.365	1.44	0.81, 2.54	0.215	1.23
Birth weight z-score	1.50	0.99, 1.11	0.092	1.04	0.98, 1.10	0.158	1.07	1.01	0.94, 1.08	0.822	1.00	0.93, 1.06	0.914	1.08	1.14	1.04, 1.24	0.004	1.17	1.07, 1.28	0.001	1.09
**7-10 years Weight status**																					
Underweight and normal weight	1			1				1			1				1			1			
Overweight and Obesity	10.71	8.26, 13.90	<0.001	10.17	7.78, 13.08	<0.001	1.29	8.00	5.94, 10.78	<0.001	7.91	5.85, 10.70	<0.001	1.34	17.12	10.26, 28.57	<0.001	17.06	10.21, 28.51	<0.001	1.24
BMI z-score	1.94	1.64, 2.29	<0.001	2.04	1.84, 2.26	<0.001	1.07	1.76	1.51, 2.07	<0.001	1.93	1.73, 2.14	<0.001	1.07	2.75	2.40, 3.15	<0.001	2.94	2.53, 3.41	<0.001	1.04

BMI: body mass index; RR: risk ratio; CI: confidence interval; VIF: variance inflation factors

*The model adjusted for child's breastfeeding status, age, area of residence, the highest degree of parental education, child's duration of sleep at night at 11-13 years, physical activity level at 11-13 years

Supplement Table S2. Crude and adjusted risk ratios for central obesity in early adolescence (ages 11–13 years) using GEE with a Poisson distribution

	Central Obesity																				
	Total							Boys							Girls						
	Crude			Adjusted*				Crude			Adjusted*				Crude			Adjusted*			
	RR	95% CI	P	RR	95% CI	P	VIF	RR	95% CI	P	RR	95% CI	P	VIF	RR	95% CI	P	RR	95% CI	P	VIF
**Birth**																					
Birth weight																					
<3.0 kg	1			1				1			1				1			1			
3.0-3.9 kg	0.91	0.67, 1.25	0.569	0.92	0.67, 1.25	0.596	1.23	1.08	0.71, 1.64	0.726	1.06	0.70, 1.61	0.772	1.17	0.81	0.51, 1.29	0.38	0.81	0.52, 1.28	0.379	1.30
≥4.0 kg	1.10	0.83, 1.44	0.507	1.08	0.82, 1.43	0.567	1.24	1.08	0.76, 1.54	0.662	1.03	0.72, 1.47	0.891	1.27	1.11	0.71, 1.71	0.652	1.19	0.77, 1.28	0.438	1.23
Birth weight z-score	1.05	1.00, 1.10	0.062	1.05	1.00, 1.10	0.061	1.07	1.04	0.98, 1.11	0.223	1.03	0.97, 1.10	0.295	1.08	1.06	0.98, 1.15	0.142	1.09	0.99, 1.19	0.073	1.09
**7-10 years Central obesity status**																					
No	1			1				1			1				1			1			
Yes	4.05	3.30, 4.98	<0.001	4.12	3.36, 5.04	<0.001	1.41	4.15	3.20, 5.39	<0.001	4.13	3.20, 5.35	<0.001	1.39	4.07	2.93, 5.65	<0.001	4.15	3.00, 5.73	<0.001	1.48
Waist circumference z-score	2.56	2.30, 2.85	<0.001	2.58	2.31, 2.87	<0.001	1.07	2.64	2.30, 3.04	<0.001	2.71	2.34, 3.14	<0.001	1.04	2.55	2.15, 3.03	<0.001	2.53	2.13, 3.01	<0.001	1.14

RR: risk ratio; CI: confidence interval; VIF: variance inflation factors

*The model adjusted for child's breastfeeding status, age, area of residence, the highest degree of parental education, child's duration of sleep at night at 11-13 years, physical activity level at 11-13 years

Supplement Table S3. Adjusted odds ratios for overweight/obesity and central obesity in early adolescence (11–13 years) in total adolescents, with interaction by sex, using Generalized Estimating Equations

	Overweight and General Obesity*				Central Obesity*			
	AOR	95% CI	P	VIF	AOR	95% CI	P	VIF
**Birth**								
Birth weight								
<3.0 kg	1				1			
3.0-3.9 kg × Girl	0.79	0.30, 2.09	0.635	2.97	0.69	0.31, 1.56	0.374	2.97
≥4.0 kg × Girl	1.3	0.57, 2.99	0.531	2.13	1.08	0.51, 2.29	0.845	2.13
Birth weight z-score								
Birth weight z-score × Girl	1.2	1.02, 1.42	0.032	1.8	1.05	0.90, 1.23	0.526	1.8
**7-10 Weight status**								
Underweight and normal weight/Central obesity								
No	1				1			
Yes × Girl	1.36	0.60, 3.05	0.463	2.04	0.8	0.44, 1.48	0.481	2.93
BMI z-score/ Waist circumference z-score								
BMI z-score/ Waist circumference z-score × Girl	1.2	0.70, 2.06	0.506	1.78	0.76	0.49, 1.19	0.229	2.02

BMI: body mass index; AOR: adjusted odds ratio; CI: confidence interval; VIF: variance inflation factors

*The model adjusted for child's breastfeeding status, age, area of residence, the highest degree of parental education, child's duration of sleep at night at 11-13 years, physical activity level at 11-13 years
